# Tau Oligomers Neurotoxicity

**DOI:** 10.3390/life11010028

**Published:** 2021-01-06

**Authors:** Grazyna Niewiadomska, Wiktor Niewiadomski, Marta Steczkowska, Anna Gasiorowska

**Affiliations:** 1 Nencki Institute of Experimental Biology, Polish Academy of Sciences, 02-093 Warsaw, Poland; 2 Mossakowski Medical Research Centre, Polish Academy of Sciences, 02-106 Warsaw, Poland; wniewiadomski@imdik.pan.pl (W.N.); msteczkowska@imdik.pan.pl (M.S.); anigas@wp.pl (A.G.)

**Keywords:** tau oligomers, neurotoxicity, tauopathies, genome, mitochondria, synapses, signal transduction, protein degradation

## Abstract

Although the mechanisms of toxic activity of tau are not fully recognized, it is supposed that the tau toxicity is related rather not to insoluble tau aggregates but to its intermediate forms. It seems that neurofibrillar tangles (NFTs) themselves, despite being composed of toxic tau, are probably neither necessary nor sufficient for tau-induced neuronal dysfunction and toxicity. Tau oligomers (TauOs) formed during the early stages of tau aggregation are the pathological forms that play a key role in eliciting the loss of neurons and behavioral impairments in several neurodegenerative disorders called tauopathies. They can be found in tauopathic diseases, the most common of which is Alzheimer’s disease (AD). Evidence of co-occurrence of b-amyloid, α-synuclein, and tau into their most toxic forms, i.e., oligomers, suggests that these species interact and influence each other’s aggregation in several tauopathies. The mechanism responsible for oligomeric tau neurotoxicity is a subject of intensive investigation. In this review, we summarize the most recent literature on the damaging effect of TauOs on the stability of the genome and the function of the nucleus, energy production and mitochondrial function, cell signaling and synaptic plasticity, the microtubule assembly, neuronal cytoskeleton and axonal transport, and the effectiveness of the protein degradation system.

## 1. Introduction

Neurodegenerative diseases share many common pathomechanisms, the most prevalent of which is the death of specific populations of neurons as a result of disturbed protein metabolism, which leads to the deposition of pathological products of their metabolism in the central nervous system. Many data indicate that protein conformation disorders are the mechanism leading to both direct neurotoxicity and insufficiently effective elimination processes of these proteins. As a result, the products of their metabolism are deposited both in the intercellular space and inside neurons. Among intracellular aggregates, the most common are filamentous tangles (NFT, neurofibrillar tangles) [1], which are pathomorphological deposits of the tau protein resulting from disturbance of post-translational processes of this protein in the nerve cell. NFTs were first identified and described in the pathology of Alzheimer’s disease [2], but they are also present in the pathology of many other dementia diseases, which are collectively known as tauopathies.

In its physiological state, tau is a protein with many functions that can have both positive and negative consequences for the cell [3]. This is due to its ability to form complexes with many proteins and other elements in the cell, and specifically, it may participate in many signaling pathways that determine cell functioning and survival [4,5]. Conversely, disturbances related to (i) alternative splicing of the tau gene, (ii) abnormal interactions between individual post-translational modifications, (iii) the formation of pathological tau aggregates, (iv) interactions with elements of the cytoskeleton, and (v) the coexistence of pathologies of other proteins forming amyloids lead to the appearance of pathological forms of the tau protein [6]. It was assumed that NFTs were the cause of neuronal toxicity, since they correlate very well with cognitive decline and neuronal loss [7]. However, in some animal models overexpressing tau, neurodegeneration has been demonstrated in the absence of NFT pathology [8]. The formation of insoluble NFTs was suggested rather to be a protective mechanism than a necessary precursor of the neuronal death in tauopathies [9]. Based on the obtained data, the researchers suggest that the presence of tau protein deposits in the form of NFT not only does not have a toxic effect on cells but is likely to repair damage caused by free radicals in the course of neurodegenerative diseases. Overexpression of the tau protein in neurons reduces the level of oxidative stress and prevents apoptosis, regardless of the tau hyperphosphorylation process taking place in the cell [10]. The obtained data indicate that phosphorylation of the tau protein may be a specific cell response to oxidative stress and is neuroprotective [11].

A growing body of experimental data has shown that tau in the nervous system can occur in many forms that perform physiological functions, but it can also display a pathological activity [12]. Forms of tau that are probably not toxic are monomers (≈60 kDa), straight filaments, paired helical filaments (PHF), neurofibrillary tangles (NFT), and ghost tangles [13]. In contrast, some types of tau dimers/trimers (120–180 kDa), small soluble oligomers (300–500 kDa), and granular tau oligomers (~1800 kDa) possibly display toxic activities [14,15,16,17].

In the pathology of Alzheimer’s disease (AD), a recently debated issue has become to explain the role of tau oligomerization in the disease pathology. Tau protein oligomers are characterized by a secondary β-sheet structure and contain 3 or 4 repeats of the microtubule binding domain, and after reaching a size greater than 20 nm, they begin to aggregate and, as a result, form fibrillar forms [18,19]. Tau oligomers are composed primarily of monomeric or dimeric subunits of the highly phosphorylated or pathologically truncated tau protein [20]. However, assembly of two distinct dimers and higher-order oligomers from full-length tau was also observed [21]. Granular tau oligomers consisting of approximately 40 tau protein molecules have also been identified in the brain tissue of AD patients. The results of the conducted research indicate that granular oligomers appear inside neurons even before the formation of PHF, because their presence is detected in the earliest stages [2] of Alzheimer’s disease [17,22].

Similarly, the role of tau monomers may be ambiguous, as their potential toxicity cannot be ruled out when they undergo post-translational modifications (PTMs) in the protein maturation process. Several studies demonstrated that AD-associated PTMs of tau impairs neuronal function, structure, and viability [23]. When tau composed mostly of monomers is injected into the hippocampus, it reduces the number of synapses, causes a loss of synaptic vesicles, and exerts detrimental effects on the morphology and connectivity of newborn granule neurons of dental gyrus, and these effects correlate with impaired behavior [24]. In addition, tau modifications suppress compensatory responses to mitochondrial stress and thus lead to numerous metabolic disturbances and reduced energy production [25]. It seems that the mechanism of toxicity of modified monomers is different from that of oligomers. The intracerebral injection of toxic monomers cleaves endogenous tau by activating calpain and consequently triggers apoptotic neuronal death, whereas oligomers induce auto-aggregation of the endogenous tau by conformational changes of the protein regardless of its phosphorylation [23]. In addition to oligomers and modified monomers, also, truncated tau containing the N-terminal domain exhibits toxic properties, causing disrupted Ca^2+^-dependent glutamate release, perturbation in K^+^-evoked calcium dynamics, deterioration in presynaptic terminals, neuritic degeneration, microtubule collapse, and reduction of mitochondrial density [26].

The high toxicity of TauOs may be related to their presence in the extracellular space and their propagation through nerve connections [27]. The presence of tau in the extracellular space is the result of the leakage of tau deposits from inside the degenerating neuron. Tau can then exist as single molecules as well as in the form of low complexed aggregates/oligomers [28]. A possible mechanism for the appearance of tau in the extracellular space is the secretion of tau aggregates in micro vesicles such as exosomes and ectosomes [29,30] and its release by exocytosis in a chaperone-dependent manner. There is also evidence that most of the extracellular tau is not associated with vesicle secretion [31]. Truncated tau or oligomeric tau may also propagate between cells by tunneling nanotubes [32,33]. Once tau appears in the extracellular space, it may undergo endocytosis by surrounding cells and influence their functioning [34,35]. The results of the experiment of Pampuscenko et al. [36] showed that the extracellular self-assembly dimers–tetramers of the 1N4R tau isoform (one extra exon in the N-terminal domain and four tubulin binding motifs), but not monomers, caused neuronal death in mixed neuronal/glial cell cultures. In contrast, monomeric and pre-aggregated tau peptide with 4R repeats but lacking the N-terminal fragment did not reduce the viability of the cells, suggesting that tau oligomers’ neurotoxicity might be dependent on the presence of the N-terminal fragment in the molecule.

It is possible that the toxic effect of the extracellular tau species is based on their interaction with M1 and M3 muscarinic membrane receptors [37,38]. As a consequence, there is an increase in intracellular calcium [Ca^2+^] and cell die due to excitotoxicity [27,39]. Several forms of tau were tested in these studies such as recombinant human tau (isoform with three tubulin binding motifs and two extra exons in the N-terminal domain), tau fragment 2N, which contains the amino-terminal half of the tau protein tau fragment 3RC, which contains the three tubulin-binding motifs and the carboxyl-terminal region, and tau fragments 4R, 3R, i.e., four or three tubulin-binding motifs. It was shown that mainly the tau fragment 3RC containing the C-terminal region and comprising residues 391 to 407 of the tau molecule is responsible for the excessive influx of calcium ions into the neuroblastoma cells. Recent reports suggest that the neurotoxic effect of extracellular tau interaction with muscarinic receptors is dependent on the activity of alkaline phosphatase [40].

**Figure 1:**
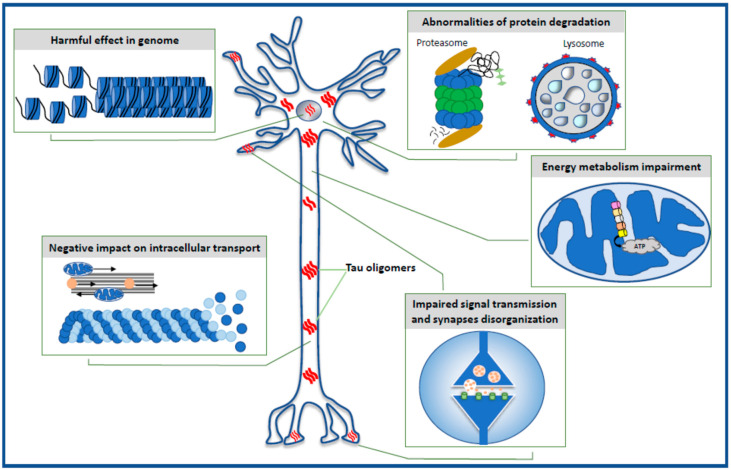
Schematic illustration of intraneuronal processes affected by toxic tau oligomers.

The data reviewed in the present paper highlight the toxic effect of oligomeric forms of the tau protein on selected intraneuronal functions such as the stability of the genome and the function of the nucleus, energy production and mitochondrial function, cell signaling and synaptic plasticity, the microtubule assembly, neuronal cytoskeleton and axonal transport, and the effectiveness of the protein degradation system (Figure 1).

## 2. Harmful Effects in Genome

The presence of tau protein in the nucleus of neurons [41,42] and the capacity of tau protein to form protein–DNA complexes have been reported [41,43,44,45,46]. It has been proven that tau-interacting DNA regions are positioned at regular intervals in the structure of the chromosome. Tau protein plays a role also in the maintenance of genomic stability [47], protection of RNA and DNA from damage induced by oxidative stress [41,42,48], and in sustenance of the dense chromatin structure [49,50]. Dysfunctional nuclear tau may disrupt heterochromatin organization, leading to cell cycle re-entry, which is fatal to neurons, and dysregulate gene expression and RNA transcription, giving rise to altered protein synthesis. Post-translational modifications or conformational changes of the tau molecule can modulate its nuclear translocation and function [51]. An aberrant modification of tau in diseases such as AD could alter its function and enhance genome vulnerability and neurodegeneration [51].

Mansuroglu et al. [50] studied the physiological role of tau protein in maintaining the neuronal genome structure and organization, namely, the role of tau protein in the organization of pericentromeric heterochromatin (PCH) DNA regions. PCH contains very dense chromatin substructures rich in epigenetic factors, such as the trimethylated form of lysine 9 of histone H3 (H3K9me3) and the protein heterochromatin 1α (HP1α), which regulate genome expression and stability [52,53,54]. The studies were performed on primary cultures of neurons obtained from wild-type (WT) or tau-deficient (KOTau) mice. In WT mouse neurons, the tau protein was found within or in the immediate vicinity of PCH and interacting with major PCH satellite sequences [50]. Mansuroglu et al. [50] also showed that in the neurons of WT mice, H3K9me3 and HP1α were distributed in PCH in the form of regular clusters. This cluster distribution of H3K9me3 and HP1α was disrupted in the neurons of KOTau mice, although the total amount of H3K9me3 and HP1α remained unchanged compared to WT mice. Under heat stress condition, the disruption of PCH organization in the neurons of KOTau mice was associated with a high degree of DNA breaks accumulated mainly in PCH sequences [50]. At the same time, the loss of clustered distribution of H3K9me3 observed in KOTau neurons affected the localization of one of the histone proteins phosphorylated on serine 139 residue (γH2AX) and made KOTau neurons unable to repair PCH–DNA breakage induced by stress [50]. Interestingly, an induced overexpression of hTau in the nuclei of KOTau neurons restored the clustered distribution of H3K9me3 that was lost due to primary tau protein gene silencing. This observation confirmed the regulatory role of nuclear tau protein in the clustering distribution of H3K9me3 and HP1α in the structure of PCH.

The expression of γH2AX is an early cellular response to the induction of DNA double-strand breaks and is used as a highly specific and sensitive molecular marker to monitor the initiation of DNA damage [55]. Similar changes in γH2AX expression were reported in neurons from AD brains [56,57]. Mansuroglu et al. [50] suggested that changes in the structure of PCH, such as the disturbed localization pattern of H3K9me3, are partially responsible for the increased expression of heterochromatically silenced genes reported in the hippocampal neurons of AD patients [49].

H3K9me3 and γH2AX were detected not only in the nucleus but also as scattered small clusters in the cytoplasm of KOTau neurons [50]. This cytoplasmic distribution of H3K9me3 was also observed in neurons of AD patients and was co-localized with AT8 antibody staining, which detects phosphorylated forms of tau [58]. This may suggest that post-translational tau modifications affect the translocation of the protein between the nucleus and the cytoplasm and alter its physiological function.

Loss of tau nuclear physiological function may be caused by its oligomerization [59]. The physiological inhibitory effect of tau on gene expression has been demonstrated under heat shock conditions (HS) [60]. Tau appears to protect tau-interacting genes from deregulation that can occur during the HS-induced overexpression of these genes. The increased presence of pathological TauOs in the nuclear compartment of neurons was correlated with their inability to bind to DNA and their incapability to repress Tau-interacting genes [59]. The transcriptionally repressing role of Tau protein was augmented in neurons from the hippocampal CA1 region of THY-Tau22 mice, where a nuclear accumulation of pathological oligomerized forms of Tau protein was detected [59]. Tau oligomers were recognized by antibodies TOC1, AT100 for tau phosphorylated at residues 212 and 214, and Tau1au for unphosphorylated tau between residues 189 and 207. In this study, nuclear accumulation of pathological oligomerized forms of tau resulted in significant deregulation of the *Dlg2* gene (coding Discs Large Scaffold Protein 2) expression in neurons from the CA1 region of THYTau22 mice. *Dlg2* gene is connected with long non-coding (lnc)RNA and contains the highest number of tau-interacting sites, and it codes a key scaffolding protein for postsynaptic membranes (postsynaptic density protein 93-PSD-93) [61]. Deficiency of this gene product may cause disturbances in the clustering of membrane receptors, the permeability of ion channels, and associated synapse signaling.

Tau oligomers can interact with p53, which is a transcription factor involved in many processes such as apoptosis [62], DNA damage repair [63], and cell cycle control [63]. Farmer et al. [64] proved that the p53 protein and TauOs (recognized by the T22 antibody) interact in the neurons of AD patients and a mouse model associated with AD (Tg2576/Tau P301L). The evidences presented in the study by Farmer et al. [64] suggest that the interaction between p53 and TauOs may be due to protein misfolding. In the p53 molecule, the DNA-binding domains, the N-terminal domain and the C-terminal base domain, were in a state of intrinsic disorder [65,66] and therefore prone to misfolding. Tau is also intrinsically disordered [67] and therefore contained many aggregation-prone regions that could interact with p53. It is known that p53 is normally associated with microtubules [68]. Thus, the proximity of p53 and tau may increase the likelihood of p53 interaction with tau, also at an earlier stage of pathology, when TauOs are initially formed. The interaction of p53 and TauOs leading to the aggregation of both proteins inhibits nuclear import of the p53 and causes loss of its function of the transcription factor [64]. Lasagna-Reeves et al. [69] showed that aggregated p53 cannot bind to DNA, confirming that aggregation leads to a loss of p53 function. Moreover, the structure of the nuclear membrane and its pores can be affected by pathological forms of tau [70]. This may hinder p53 transport into the nucleus and cause its accumulation in the cytoplasm, which has been observed both in neurons of AD patients and tau overexpressing mice [64,71].

Benhelli-Mokrani et al. [59] showed that the half of the tau-interacting DNA sequences are positioned within non-protein coding intergenic regions. They include DNA sequences coding long non-coding (lnc)RNAs and DNA regions comprising an AG-rich motif [59]. lncRNAs exhibiting an aberrant expression rate were observed in AD [72]. AG-rich motifs are the binding sites of GAGA factor, which is a transcriptional activator involved in regulation of the chromatin structure. Repeated AG-rich GAGA motifs are regularly dispersed throughout the genome and regulate its 3D structure through DNA boundaries and loops organization [73]. Loops contains actively transcribed genes and emanate from condensed chromatin. Boundaries belong to a special class of architectural elements that regulates chromatin compartmentalization. Boundaries are distributed throughout the genome and are the ubiquitous building blocks of chromosomes [74]. GAGA motifs associate inactive chromosomal domains to the inner nuclear lamina [75] and regulate genome compartmentalization and chromatin silencing in mammalian cells. The fact that tau-interacting DNA regions include a recurrent AG-rich GAGA-like motif displays a potential role for tau protein in nuclear organization [59]. Lamina dysfunction and heterochromatin disruption was connected with tau pathology [76,77]. These might be caused by disturbance in TauOs interaction with AG-rich GAGA motifs and could play an active role in tauopathies, as demonstrated by the studies of Benhelli-Mokrani et al. [59]. They analyzed RNA extracted from the cortex and hippocampus from 17 m KOTau mice and WT littermates, and the hippocampal CA1 region from 6 m THY-TAU22 mice and WT littermates. These studies confirmed the nuclear presence of pathological oligomerized forms of tau detected with Tau1, TOC1, and AT100.antibodies.

The nucleotoxic activity of TauOs may be related to the dysfunction of the Musashi family proteins (MSI1 and MSI2). MSIs belong to RNA-binding proteins involved in the transcription and post-transcriptional regulation of genes [78]. Montalbano et al. [79] observed oligomeric forms of MSI1 and MSI2 in the cytoplasm and in the nuclei of cortical neurons of AD, ALS (amyotrophic lateral sclerosis), and FTD (frontotemporal dementia) patients, where they were localized with oligomeric species of tau. Tau oligomers were identified with the TOMA2 antibody (anti-oligomeric tau monoclonal antibody). Montalbano et al. [79] also conducted studies on two iHEK (immortalized human epidermal keratinocytes) cell lines that express human wild-type tau or the form of tau containing the P301L mutation. In the P301L line, increased associations were observed between MSI2, toxic TauOs, Histone3, and Lamin (nuclear fibrous protein). This large complex can damage the chromatin structure and disarray its compartmentalization in the nucleus. In cells overexpressing the mutant form of tau, a significant decrease in the levels of the proteins LaminB1 and LaminA/C in the nuclear membrane was observed. This was accompanied by a general downregulation of nuclear membrane linkers such as emerin, LaminB receptor, companion lamellar protein 3 Man1 containing the polypeptide 2-emerin domain, and the nuclear envelope type II transmembrane protein Nesprin2. This resulted in instability of the nuclear membrane. Chromatin linkers such as heterochromatin 1 protein (HP1), plaque 2-associated polypeptide (LAP-2), and autointegration barrier factor (BAF) were also reduced.

Disruptions in the structure of the nuclear membrane and the pore system caused by pathological forms of tau were also described in the studies by Montalbano et al. [79]. Toxic tau influenced nucleocytoplasmic import, preventing substances from penetrating into the nucleus through the nuclear pore complex. It has been shown that TauOs can bind to phenylalanine/glycine-rich nucleoporins Nup98 and Nup62 and affect the permeability of the nuclear pore complex. The authors [79] also observed a decreased level of Nup50 and Nup153 nucleoporins in the nuclear fraction and at the same time their increased level in the cytoplasm of P301L tau iHEK cells. Thus, tau oligomers by interacting with nuclear transport receptors can seriously dysregulate intra-nuclear transport.

Pathogenic tau directly contributes to the depletion of cAMP response element binding protein (CREB), which is a major regulator of gene transcription critical to neuronal survival, neural plasticity, learning, and memory [80]. A significant decrease in the level of CREB and phospho-CREB was noted in *post mortem* human AD brain tissue [81] and in cultures of hippocampal neurons derived from tau transgenic mice [82]. Mahoney et al. [83] investigated whether pathogenic forms of tau influenced nuclear Ca^2+^ levels, which are critical for CREB-dependent gene expression. The observations were carried out in neurons derived from induced pluripotent stem cells (iPSCs) of patients with sporadic AD and in the *Drosophila melanogaster* tauopathy model expressing human tau bearing the R406W mutation [84]. TauR406W is an autosomal dominant mutation that causes frontotemporal lobe degeneration (FTLD) [85]. It was found that the neuronal expression of human transgenic tauR406W in the brain of adult Drosophila resulted in a reduction in the total and nuclear CREB protein level. It was observed that in the resting state, the level of nuclear Ca^2+^ decreased with physiological aging, and pathogenic tauR406W significantly increased the age-related loss of calcium ions from the nucleus. It has also been reported that blocking the nuclear Ca^2+^ signal enhances tauR406W-induced neuronal death, suggesting that Ca^2+^ nuclear depletion is a causal mediator of neurodegeneration in tauopathy [83].

In summary, toxic tau species including TauOs can affect the genome transcription in several ways. They can directly interact with the DNA and affect the nuclear envelope, making it unstable and hindering the transport of transcription factors by nuclear pore complexes. This results in damage to the chromatin structure, disruption of gene expression, and cytoplasmic accumulation of transcription factors that are prone to aggregation.

## 3. Impairment of Energy Production in the Neuron

Mitochondrial impairment entails disturbance in, inter alia, ATP production, regulation of lipid and calcium metabolism, removal of reactive oxygen species (ROS), and induction of cell death [86,87]. This was well described in neurons of AD brains [88,89,90] and has been connected with pathological forms of tau such as its TauOs [91]. Tau oligomers can indirectly interrupt the energy supply because of their destabilizing effect on microtubules, resulting in the impairment of the mitochondria distribution across a neuron, especially in axonal terminals and synapses where the mitochondria are transported over a very long distance [92].

The neurotoxic impact of TauOs on mitochondrial functions was studied in the hippocampus of C57BL/6 wild-type (WT) mice, whose brains (CA1 of hippocampus) were injected with oligomers (120–170 kDa) of full-length recombinant h-tau441 (2N4R) [92]. Lasagna-Reeves et al. [92] presented the co-localization of TauOs immunostained with HT-7 (antibody recognizing epitope of human tau between residue 159 and 163) with mitochondrial marker, porin, which suggested a direct interaction of TauOs with mitochondria. This may indicate that TauOs are internalized into the mitochondrial membrane in the mouse brain, which in turn may induce changes in the cohesion of the mitochondrial membrane.

Toxic tau species can negatively influence energy production. In hemispheres of mice injected with TauOs decreased level of complex I (NADH: ubiquinone oxidoreductase, a mitochondrial respiratory chain component) was observed [92]. David et al. [93] showed reduced levels of another component of the mitochondrial respiratory chain complex V (ATP synthase) in the brains of transgenic mice overexpressing mutant human tau P301L, in which also tau hyperphosphorylation was manifested.

Lasagna-Reeves et al. [92] also reported an increase in pro-apoptotic caspase-9 activation in the hemispheres of mice injected with TauOs. The authors proposed that caspase-9 activation could be stimulated by cytochrome c (cyt c) released from mitochondria because of the accumulation of TauOs in the mitochondrial membrane. Mitochondrial membranes can be permeabilized by toxic TauOs. It has been hypothesized that these oligomers interact electrostatically with anionic membrane phospholipids and penetrate into the lipid bilayer where they can be formed into pore-like structures [94,95].

The direct effect of TauOs on mitochondrial membranes was measured by Camilleri et al. [96]. A unique phospholipid, 1,3-diphosphatidyl-sn-glycerol, called cardiolipin (CL), appears to play a key role in the toxicity of TauOs to mitochondrial membranes. Under physiological conditions, the presence of CL guarantees an optimal organization of the respiratory electron transport chain in the inner mitochondrial membrane (IMM). CL is localized primarily in the areas of contact of the outer and inner mitochondrial membrane, which allows CL to diffuse from one membrane to the other. In this way, metabolites of the mitochondrial matrix and the cytosol can be easily exchanged. CL is also a fundamental component of the mitochondrial apoptotic pathway associated with the mobilization of cyt c [97,98]. The assumption that TauOs are capable of destabilizing mitochondrial membranes and that CL is involved in this process was tested in model studies. For this purpose, two types of isolated membranes were constructed with a lipid composition imitating the biophysical properties of the mitochondrial bilayer. One of these membrane constructs lacked CL. These membranes formed liposomes that were incubated in solution containing the TauOs. Channel-like structures 1–2 nm in diameter formed by the tau protein were detected in CL-rich liposomes [96]. Electrophysiological data confirmed the formation of pores or tau channels.

It is known that monovalent and divalent negatively or positively charged ions and small molecules can easily pass through nanopores of small diameter. This led to the suggestion that the perforation of the mitochondrial membrane induced by abnormal tau might cause mitochondrial edema [96]. This hypothesis was proven in the experiment of Camilleri et al. [96]. Mitochondria isolated from SH-SY5Y cells were exposed to oligomeric tau at low micromolar concentration. Significant swelling of the organelles and outflow of cyt c from the mitochondria was observed. The conclusions of the experiment by Camilleri et al. [96] were supported by the previous studies of Cieri et al. [99], who showed that tau is located in the outer mitochondrial membrane and in the intermembrane mitochondrial space.

Interestingly, not only can TauOs negatively alter mitochondrial physiology as mentioned above, but conversely, disturbances in mitochondrial metabolism such as inhibition or decoupling of the respiratory chain promote tau oligomerization [100,101]. It has been shown that these changes may result from mtDNA (mitochondrial DNA) disturbances. Cybrid cell lines containing mtDNA from AD patients, as well as cell lines characterized by chronic and acute mtDNA depletion, were found to have higher levels of total tau and TauOs and increased tau phosphorylation on serine 199 [102]. Lowering mtDNA levels can also trigger an adaptive stress response or adaptive metabolic reprogramming. For example, Weidling et al. [102] showed that cytochrome oxidase (COX) activity is proportional to mtDNA copy number. The authors also showed that dysregulation of the respiratory chain caused by mtDNA disturbances induces the accumulation of TauOs in mitochondria. In this study, TauOs were identified with the TOC1 antibody, which binds the unique tau conformation associated with soluble tau dimers and slightly larger oligomers, and the Tau12 antibody, which recognizes a linear epitope in the N-terminal (amino acids 9–18) and detects tau mono- and oligomers. Thus, a study by Weidling et al. [102] proved that mitochondrial dysfunction induced by mtDNA abnormalities can both increase TauOs level and facilitate the oligomerization of tau from monomers.

A very interesting hypothesis is proposed by Zheng et al. [103], who believe that in the early phase of tau pathology, soluble TauOs may stimulate protective processes promoting mitochondrial energy production and neuronal survival. mtDNA adheres to the IMM, from where ROS are released, so it is constantly exposed to oxidative damage. However, there is a DNA repair mechanism in the mitochondria that is classified as DNA base excision repair (BER). In neurons in which tau oligomerization is present, the mitochondrial structure changes, and the activity of DNA repair processes increases [103]. Zheng et al. [103] investigated the protective mechanism stimulated by soluble TauOs in the mitochondria of the CA1 neurons of 6-mo THY-Tau22 mice expressing human tau46 mutated at positions G272V and P301S. Toxic TauOs were detected with TOC1, phospho-Tau (Ser202, Thr205) AT8, and phospho-Tau (Thr212) antibodies. Soluble TauOs in hippocampal neurons stimulated changes promoting mitochondrial survival and homeostasis, and they enhanced the activity of DNA repair pathways. This response included the stimulation of SIRT3 (NAD^+^-dependent deacetylase sirtuin-3), a primary mitochondrial deacetylase whose expression is modulated by oxidative and metabolic stress and whose decreased level in AD is associated with mitochondrial dysfunction [104,105,106]. The increased level and activity of SIRT3 in the CA1 region of the 6-mo THY-Tau22 mice allowed the maintenance of mitochondrial function.

Tau oligomers can influence mitochondrial fusion and fission, which are processes that help eliminate abnormal mitochondria. The enhancement of mitochondrial fusion maintains mtDNA integrity and regulates the morphology and distribution of mitochondria in the cell [107]. On the other hand, the cytosolic Drp1 (dynamin regulated protein 1) protein, which is the major regulating factor for fission, has been shown to interact with hyperphosphorylated tau in the brain of Alzheimer’s patients [108]. In 6-mo THY-Tau22 mouse hippocampal cells, DRP1 expression was significantly reduced in the early phase of tauopathy, which altered the balance in favor of fusion processes and consequently promoted mitochondrial elongation and maintained mitochondrial homeostasis [103]. Moreover, the presence of TauOs in the neurons of these mice increased the level of 8-oxo-G, which is a product of oxidative DNA damage. However, at the same time, elevated levels of 8-oxo-G DNA glycosylase (OGG1), the enzyme responsible for removing 8-oxo-G were observed in these mice, which may be an element of mitochondrial protection. Another element involved in the pro-survival response triggered by TauOs-induced mitochondrial stress is polymerase β (Polβ). This polymerase is present mainly in the nucleus, but it has also been found in the mitochondria where it contributes to the integrity of mtDNA. Elevated Polβ levels were observed in the mitochondria of the neurons of the 6-mo THY-Tau22 mice. This suggests the protective relocation of Polβ from nucleus to mitochondria, which may promote mtDNA repair.

The protective mechanisms described above in early tau pathology may have an impact on the long-term survival of neurons. However, these protective mechanisms may also allow the progressive accumulation of tau aggregates in affected neurons and an increased risk of propagation of pathological forms of tau to neighboring neurons over time, as observed in AD [109].

## 4. Selective Effect of Tau Oligomers Treatment on Synaptic Integrity and Function

Since the discovery of tau mutation as a cause of FTLD, tau has been implicated in different synaptic dysfunctions, such as impaired LTP, reduced excitatory synaptic transmission, decreased level of PSD-95 and glutamate receptors, and increased levels of glutamate and excitotoxicity [110]. Usenovic et al. [111] noted that the model relevant for sporadic tauopathies, such as AD, in which the MAPT (microtubule-associated tau protein) mutations are absent would be one in which changes similar to those seen in AD would occur despite the absence of a mutation in the tau protein or lack of tau overexpression. They proposed such a model: the human forebrain neurons with active GABAergic and glutamatergic receptors, which were derived from induced pluripotent stem cells and treated with preparations of tau monomers and oligomers for 24 h. In this model, fourteen days after TauOs were applied, the number of synapses declined, basal GABA (gamma-aminobutyric acid) release moderately increased, and glutamate release remain unchanged. In addition to synaptic-related changes, they observed a host of other changes, which are similar to those characterizing AD. No such changes were observed when tau monomers were applied.

Effects of oligomeric tau treatment on synaptic integrity and function were also evident within a much shorter time frame. Lasagna-Reeves et al. [92] found in the neurons of the CA1 region of the hippocampus that the subcortical injection of TauOs reduced the levels of synaptophysin, which is a synaptic vesicle bound protein, and of septin-11, which is a protein involved in vesicle trafficking. On the other hand, the level of synapsin-1, the protein that regulates the reserve pool of synaptic vesicles, remained unchanged. These effects were observed about 30 h post-injection. The animals treated with TauOs did not distinguish the new object in the Object Recognition Task. This may mean that TauOs impair the ability to store newly acquired information, which is the kind of memory deficit occurring in early stage of AD. The injection of tau monomers and fibrillary forms neither affected the level of synaptic proteins nor caused memory deficit.

Fa et al. [112] showed that a brief exposure of mouse hippocampal slices to different recombinant human TauOs, but not tau monomers, reduced LTP as soon as 20 min after exposure to the oligomers. This finding was paralleled by the demonstration that two bilateral injections of TauOs performed shortly prior to the training into the dorsal mouse hippocampi impaired associative fear memory assessed 24 h later. The authors also demonstrated the worsening of spatial memory in a radial arm water maze after infusion of oligomeric tau.

Ondrejcak et al. [113] injected into brain ventricles soluble human recombinant tau aggregates and observed the potent inhibition of LTP at CA3 to CA1 synapses of the hippocampus, whereas no change in LTP followed the injection of tau monomers and tau fibrils. The gradual decline of LTP was observed during the 3 h period following tau injection. The effect of TauOs was prevented by the administration of anti-tau monoclonal antibody, Tau5 to the prion protein (PrP), which may suggest that the involvement of PrP in tau oligomers induced LTP reduction.

Hill et al. [114] delivered oligomeric full-length tau-441 (TauOs) in nanomolar concentration directly into mouse hippocampal CA1 pyramidal and neocortical layer V thick-tufted pyramidal cells. Within the first few minutes after intracellular TauOs delivery, the electrophysiological readouts remained unchanged, and some of them changed significantly after 40 min. The introduction of TauOs into hippocampal neurons increased the input resistance, membrane potential depolarization, and firing rate but decreased the amplitude, speed of rise, and speed of decay of action potentials. Examination of connected pairs of thick-tufted layer V neocortical pyramidal cells revealed that the delivery of TauOs into presynaptic cells impaired within a short time frame basal synaptic transmission, which was associated with short-term depression. The delivery of TauOs into postsynaptic cells impaired LTP, which may indicate that the presence of TauOs in postsynaptic cells interferes with synaptic plasticity. The delivery of tau monomers did not evoke any similar effects.

The observations presented above point collectively to TauOs being taken up by neurons from extracellular space, and once being inside the neurons, they change the functioning of synapses within a short time, even during tens of minutes. The relatively short time needed to reveal the effect of TauOs on various electrophysiological parameters, reported by Hill et al. [114], can be explained by the direct delivery of TauO into neurons, which shortened the time needed for TauO molecules to reach distal dendrites and axons, where they have been indeed clearly visible. These findings speak for the direct involvement of TauOs molecules in processes of synaptic transmission and plasticity. The common finding of reported data is the impairment of long-term potentiation (LTP). LTP is believed to be one of the cellular mechanisms that underlies learning and memory, and indeed, in the two studies [92,112], behavioral tests revealed TauOs were related to memory impairment.

The use of well-defined oligomeric forms of the tau protein in research shows that tau interacts with synaptic proteins. It plays a role in monitoring intracellular signaling pathways. The entering of pathological forms of tau into postsynaptic compartments and dendrites [110] causes a decrease in the number of synaptic vesicles [115] and reduction of synaptic proteins and dendritic spines, which in turn promotes synaptic loss [24], reduces neuronal signaling [116,117], and finally, induces memory impairment.

## 5. Negative Impact of Tau Oligomers on Microtubule Assembly, Neuronal Cytoskeleton, and Axonal Transport

Toxic tau species can interfere with microtubule stability, thus affecting the transport and distribution of organelles and signaling proteins (enzymes, receptors) in cell compartments [118]. An intracellular accumulation of tau was often accompanied by an increase in transport time and the instantaneous velocity of the vesicular cargos [119]. Mitochondria travel long distances along the microtubules to meet energy needs at synaptic junctions; therefore, tau by the inhibition of transport systems impairs energy production in neurons. Extracellular TauOs can also disrupt normal neuronal homeostasis by triggering axonal tau accumulation and the loss of polarized tau distribution as well as impairing rapid axonal transport [120,121].

The dynamics of the microtubules must be tightly controlled by the cells. Relatively small changes in the dynamic behavior of microtubules lead to apoptosis [122]. To maintain proper communication inside the neuron, microtubule dynamics must remain within a narrow range of activity, which is regulated by the tau protein.

The role of TauOs in regulating the state of dynamic microtubule instability is ambiguous. The presence of TauOs may be an important element in the physiological interaction of tau, as a MAP family protein, with microtubules, which maintains the equilibrium of the ongoing process of microtubule polymerization and depolarization [123]. On the other hand, Makrides et al. [123] suggest that the formation of tau oligomers containing amino acid substitutions related to, e.g., mutations present in some tauopathies, reduces the ability of tau to stabilize microtubule dynamics, resulting in cell death. Different composition of oligomers containing subunits of different microtubule binding domains (combinations of 4R and 3R forms) may cause the overstabilization (stiffening) or destabilization of microtubules and damage axon or dendrite transport [124]. By using a model of engineered FTDP-17 cells, Gyparaki et al. [125] demonstrated that tau forms physiological oligomers—mostly dimers and trimmers—on microtubules. However, in pathological conditions (aggregated tau Clone 4.1), tau forms small oligomeric structures, fibrillary assemblies, and branched fibrils, which are distinct from physiological oligomers and do not form connections with microtubules.

It should be assumed that the presence of abnormal TauOs not associated with microtubules may lead to the disturbance of neuron transport, especially fast axonal transport (FAT). Numerous studies using very different experimental models, such as the acellular squid axoplasm, in vitro cell models, and in vivo mouse models, have found that FAT is disrupted by a variety of pathological forms of tau [126,127,128,129]. Exposure of the N-terminal phosphatase-activating domain (PAD) in tau and tau oligomerization triggers a signaling pathway that disrupts axonal transport [130]. Ward et al. [126] postulated that in the physiological state, tau in neurons interacts with microtubules when it has a “paperclip” conformation, and the PAD domain is not exposed. This enables the activation of the PP1-GSK-3β (activation of protein phosphatase1—PP1 and subsequent dephosphorylation of Ser9 in glycogen synthase kinase-3β-GSK-3β) [127,131] signaling cascade and the proper transport of cargos by the motor protein kinesin along the microtubules. In pathological states (tauopathies), tau undergoes aggregation or post-translational modification, resulting in the unfolding of the “paperclip” conformation and the exposure of the PAD domain. This leads to the dissociation of tau from the microtubules and disrupted activation of the PP1-GSK-3β cascade. The consequence of this is an increased inhibition of anterograde FAT. Heat shock protein Hsp70 may play a repair role in these processes. Normally, it binds to monomeric tau, which has separated from the microtubules, thus preventing its aggregation. Hsp70 can also interact with TauOs to cause conformational changes, hiding the PAD domain and thereby inactivating the toxicity of TauOs.

Further studies [130] using the squid axoplasm revealed that recombinant wild-type tau aggregates (a mixture of oligomers and filaments) specifically inhibited anterograde FAT but did not affect retrograde FAT, while monomeric tau had no effect on FAT in either direction. Subsequent studies [132,133] showed that oligomeric forms of tau with increased PAD domain exposure disrupted both axonal transport and synaptic signal transmission. These studies also confirmed that modifications to monomeric tau can cause the toxicity of low-aggregated tau oligomers and multimers. Evidence has been provided to show that the pseudophosphorylation of tau on serine 422 prolongs the nucleation phase of tau aggregation and enhances dimer formation. Using vesicle motility tests in the isolated squid axoplasm, it has been shown that S422-phosphorylated tau monomers and oligomers (detected with the TOC1 antibody) inhibit not only anterograde but also retrograde axonal transport. In contrast to anterograde FAT, the mechanism of retrograde FAT inhibition is possibly independent of PAD domain exposure. In addition, it has been found that tau species isolated from human AD brain containing the pS422 modification also display PAD exposure and are in oligomeric conformations (TOC1 immunoreactive).

The in vitro studies of Swanson et al. [121] also showed that extracellular TauOs can damage the microtubule structure and induce axonal transport dysfunction. Extracellular TauOs induced intracellular tau accumulation and changed the localization of endogenous tau from its normally most abundant region, the axon, to the somatodendritic compartment. Exogenous TauOs containing 2N4R or 2N3R domains provoked the aggregation of endogenous intracellular tau much more efficiently than monomers or filamentous tau. Moreover, 2N4R oligomers interfered with the rapid axonal transport of membranous organelles along the microtubules. Tau accumulation induced by exogenous oligomers did not appear to promote microtubule fragmentation, as it was not accompanied by altered distribution of the neuron specific βIII-tubulin and MAP2 cytoskeleton integrity marker. One exception was the extracellular oligomers composed of a mixture of all six tau isoforms present in the central nervous system. Their administration resulted in fragmentation of neuronal processes stained with both anti-tau and anti-MAP2 antibodies, suggesting that these oligomeric forms may be toxic to microtubules.

Abnormal tau has been found to impair the axonal transport of peroxisomes, neurofilaments, Golgi-derived vesicles, neurotrophin receptors, APP (amyloid precursor protein), and other organelles into neurites [134,135]. Disruption of the transport of each of these organelles is dangerous to the neuron and can cause its dystrophy and even death. In particular, tau inhibits the transport of mitochondria into axons and dendrites, and this inhibition causes a severe impairment of energy production in areas of high energy demand such as synaptic connections [136]. Tau interacts with c-Jun N-terminal kinase-interacting protein 1 (JIP1), which is associated with the kinesin motor protein complex [137]. Abnormal tau sequestrates JIP1 in the cell body and impairs its transport to the axon, which disturbs the formation of the kinesin motor complex and impacts the kinesin-driven anterograde transport of mitochondria [138].

Proofs of different toxic impacts of abnormal tau on mitochondria have been derived mainly from in vitro and animal studies. Most studies look at abnormal post-translational modifications of tau or protein forms containing mutations that characterize neurodegenerative diseases. Important impairment in anterograde (but not retrograde) transport of mitochondria along the axon has been demonstrated in K3 mice expressing human K369I mutant tau [138]. The reduction of mitochondrial content in neurites and the perinuclear clustering of mitochondria was observed in rTg4510 mice expressing a repressible *form of* human tau containing the P301L mutation that has been linked with familial frontotemporal dementia [139]. In turn, the KI-P301L mice with the knock-in of human tau gene with the P301L mutation showed a reduced number of axonal mitochondria, an enlarged volume of motile mitochondria in the axons, and a decreased binding of tau to microtubules [140]. In in vitro studies, induced pluripotent stem cells (IPSCs) derived from frontotemporal dementia (FTD) patients bearing the R406W tau mutation were characterized by a reduced number of axonal mitochondria and an increase in the retrograde transport of these organelles [141,142]. On the other hand, in the cells of the same lineage, but carrying the N279K and P301L tau mutation, decreased anterograde transport of mitochondria was observed [141]. The changes in mitochondrial transport were associated with tau hyperphosphorylation and the cytoplasmic presence of tau aggregates detected with the AT100 anti-PHF phospho-tau antibody.

However, there are no reports directly showing the influence of TauOs on the transport of mitochondria in the compartments of nerve cells.

## 6. Defective Protein Degradation

The pivotal role in the strategy of neutralizing pathological forms of tau is played by protein quality control (PQC) system, consisting of the ubiquitin proteasome system (UPS) and autophagy. UPS primarily degrades short-lived proteins and may degrade soluble neurotoxic tau forms [143]. As for tau aggregates, it is believed that the proteasome is incapable of degrading them because its narrow chamber requires the development of a protein in order to digest it, which in the case of a highly aggregated tau may be difficult [144].

Many lines of evidence support the existence of AD-specific impairments of protein degradation systems. For instance, ubiquitin in free form or conjugated with protein was found in the senile plagues and in the NFT of AD patients [145]. The ubiquitin conjugated with PHF at sites localized to the microtubule-binding region of tau was also reported by Morishima-Kawashima et al. [146]. A non-PHF-related decline of UPS performance was described by Keller et al. [147] and López Salon et al. [148] in brains of AD patients. However, the inhibitory effect of PHF on proteasome activity was reported by Keck et al. [149]. In the brains of AD patients, the amount of PHF-tau co-precipitated during proteasome immunoprecipitation strongly correlated with the proteasome activity. Furthermore, the in vitro incubation of proteasomes with PHFs isolated from AD brains resulted in the inhibition of these proteasomes activity. Enhancing the UPS activity improved cognitive status in animal models of AD. However, at present, there is a scarcity of data clearly proving that tau aggregates impair the functioning of the proteasome.

It has been shown that both phosphorylated and non-phosphorylated tau could be degraded by UPS, i.e., with the help of ubiquitin, the 26S proteasome, and in an ATP/Mg^2+^-dependent manner [150]. Natively unfolded tau may be degraded by the 20S proteasome in a ubiquitin-independent way [151]. Tau, as other proteins, in order to be processed by the proteasome, has to be ubiquitinated by an E3 ligase, and one of them is CHIP (C terminus of HSC70-Interacting Protein). Shimura et al. [152], using transfected COS7 cells (fibroblast-like cell lines derived from monkey kidney), found that the CHIP–Hsc70 complex conjugates ubiquitin specifically to hyperphosphorylated but not to unphosphorylated tau. Due to this reaction, soluble phosphorylated tau is sequestrated into insoluble aggregates, and the cell is rescued. Based on their result, the authors believe that the accumulation of soluble p-tau is toxic, whereas the presence of insoluble ubiquitinated phosphorylated tau is not. Furthermore, they hypothesize that the CHIP-mediated ubiquitination of p-tau protects cells in two ways, by directing p-tau to the proteasome for degradation or by turning it into insoluble aggregate. In line with the pro-degradative role of CHIP, its level inversely correlated with the level of insoluble tau in AD brains [153].

The p-tau proteasomal degradation with the help of the CHIP–Hsc70 complex may be significantly more efficient when the activity of Hsp90 (heat shock protein 90), which aimed to dephosphorylate and refold p-tau, is inhibited [154]. The chaperone Hsp90 supports the folding/refolding and stability of misfolded protein, which is generally beneficial; however, it may help stabilize toxic protein aggregates, thus promoting the accumulation of their aggregates in neurodegenerative diseases. Luo et al. [155] showed, using cell cultures and mouse models, that Hsp90 allowed and sustained the accumulation of toxic Tau but not WT Tau and that the inhibition of Hsp90 led to the elimination of mutant tau and to the solubilization of tau aggregates. In AD brain, abnormally truncated tau at Asp(421) (tauDeltaC) is being found. This form of tau is prone to form fibrillary aggregates. It may be removed predominantly by macroautophagy after binding CHIP and subsequent ubiquitylation [156]. The oral gavage of sulforaphane, an herbal isothiocyanate, reduced the level of tau and phosphorylated tau in a triple transgenic mouse model of AD (3 × Tg-AD) but did not reduce the mRNA expression of tau. Sulforaphane treatment upregulated CHIP and Hsp70 and ameliorated memory deficits [157].

Another chaperone, HspB1, belongs to ten mammalian small heat shock proteins (sHsps), which is a class of molecular chaperons that typically associate early with misfolded proteins, and their interactions with client proteins are ATP-independent, in contrast to later steps of chaperone–client protein interactions [158]. It was found upregulated in samples of AD brains; however, increased levels of HspB1 in cell culture may lead to tau phosphorylation and cell cycle reentry [159]. HspB1 can bind directly to tau and delay early tau aggregation, but it does not prevent the ongoing fibril elongation [160,161]. Wild-type HspB1 overexpression but not its perpetually pseudo-phosphorylated variant reduced the tau level and spared LTP in a mouse model of tau aggregation [160].

Autophagy can be divided into chaperone-mediated autophagy (CMA), endosomal microautophagy (e-MI), and macroautophagy [162]. Macroautophagy is the major cellular mechanism responsible for removing protein aggregates, such as insoluble tau aggregates [163], long-lived proteins, damaged organelles, and pathogens, whereas CMA [164] and e-MI [165] may degrade soluble neurotoxic forms of tau. It is of interest that incompletely cleaved tau fragments crossing the lysosomal membrane during CMA may promote the formation of TauOs, which may act as precursors of aggregation and may interfere with lysosomal functioning [164].

Immature autophagic vacuoles filled with undigested or partly digested proteins were identified in the swollen or dystrophic neurites of Alzheimer patients’ brains [166]. The progressive accumulation of these vacuoles in AD brains, as well as of autophagosomes, multivesicular bodies, multilamellar bodies, and cathepsin-containing autolysosomes was described by Nixon RA [167] and Menzies et al. [168]. These findings may be explained by multiple defects concerning the maturation of autophagosome, their transport, and fusion with lysosomes. Taken together, these changes may be regarded as yet another hallmark of AD [169], as in healthy neurons, autophagosomes are rare [170]. The above described findings suggests that the deficient proteolysis due to impairment of the autophagy–lysosome pathway may be a reason for the accumulation of Aβ and tau in AD [171,172]. At present, the causative role of impaired autophagy in AD may be only hypothesized, as this impairment may be a result of other defective processes present in AD. For instance, in transgenic mice expressing human Tau with FTDP-17-like mutations, lysosomal aberrations were observed [173].

In the case of macroautophagy, items destined to degradation are tagged by chaperones and transferred to autophagosomes. In case of the distal parts of axons, autophagosomes are produced there and transported to the soma in a retrograde fashion, where they fuse with lysosomes, forming autolysosomes in which the degradation of their content is completed [174,175]. More recently, it has been shown that soma-derived degradative lysosomes are transported in an anterograde way from soma into distal axons, where they fuse with autophagosomes, forming degradative autolysosomes, some of which move back to soma. By knocking down Arl8, a lysosomal kinesin adaptor, the delivery of lysosomes to axonal terminals was impaired, and an aberrant accumulation of autophagosomes in distal part of axons was observed [176]. Both anterograde and to a lesser extent retrograde transport along microtubules is influenced by microtubule-associated tau [177]; thus, it is likely that autophagosome transport in neurites may be influenced, also adversely, by tau.

It is evident that although impaired autophagy may not be the primary cause of tauopathies, disturbing the autophagy process can induce tauopathy–like changes. In mice, deficiency of the essential autophagy-related gene 5 (Atg5) or Atg7 caused progressive neuronal degeneration, severe axonal swelling and atrophy, abnormal accumulation of intracellular proteins, and the development of numerous aggregates and inclusions [178,179]. Disrupted autophagy led to the formation of TauOs and insoluble aggregates. Induction or enhancement of the autophagy process may limit the production of these tau moieties [180,181]. Taken together, these findings raise the likely possibility of a vicious circle, whereby inefficient macroautophagy raises the level of unphysiological tau forms, which in turn further impedes macroautophagy.

Lysosomal proteolytic failure, whether primary or induced, may play a key role in the development of neurodegeneration in AD [182]. If therapeutic enhancement of autophagy is considered, the timing of its induction may be essential. For instance, the enhancement of autophagy before the development of AD-like pathology reduced the levels of soluble tau and number of immature amyloid plaques in 3xTg-AD mice. However, once the mature tangles and plaques developed, the induction of autophagy did not ameliorate the cognitive deficits present in these mice [183]. Lipinski et al. [184] demonstrated that autophagy in the healthy human brain is transcriptionally downregulated with age, whilst, surprisingly, it is upregulated in the brains of AD patients. Bordi et al. [185] analyzed hippocampal CA1 neurons from early and late-stage AD subjects’ brains and from nondemented controls and found an upregulation of autophagy-related genes, beginning at early AD stages. This was reflected in increased autophagosomes and lysosomes formation. However, they observed decreased autophagy flux due to deficient substrate clearance, which was paralleled by the expansion of autolysosome size. They suggest that a sustained induction of autophagy combined with a progressively declining lysosomal clearance of substrates leads to neuritic dystrophy as a result of the autophagic pathology.

Thus, it may be important both to enhance the defective lysosomal proteolysis and induce autophagy. Activation of transcription factor EB (TFEB) may allow accomplishing these goals [186]. TFEB is a master activator for the expression of lysosome-related and autophagy-related genes. Curcumin analog C1 (C1) is a small molecule that activates TFEB by direct binding [187]. C1 activates the autophagolysosome and reduces tau aggregates in mice models. It is currently being tested in AD Phase II clinical trials. Currais et al. [188] found that fisetin, an organic flavonoid, reduces pathological tau and alleviates learning and memory deficits in mice AD models. These effects were attenuated by inhibitors of the autophagy–lysosome pathway. Fisetin inhibits mTORC1 (mammalian/mechnistic target of rapamycin complex 1), which in turn activates TFEB and Nrf2 (nuclear factor erythroid 2-related factor 2); both these transcription factors are important for autophagy induction. At present, fisetin is tested in AD Phase II clinical trial NCT02380573 [189].

The harmful tau protein can be the subject of targeted proteolysis, which is a technique helping to remove defective proteins in the cell. Proteolysis-targeting chimeras (PROTACs) are the molecules comprised of a ligand targeting the client protein connected via a linker with a ligand targeting an E3 ubiquitin ligase. PROTAC induces the degradation of the client protein by targeted ubiquitination, leading to its proteolysis in proteasome S26. This technology was initially described by Sakamoto et al. [190] and, among others, it was adopted to target tau. There are over 600 E3 ligases in humans, some of which have been used in the anti-tau PROTAC constructs [191].

Chu et al. [192] synthesized several PROTAC molecules that recognize the tau protein, one of them called TH006, which binds the VHL E3 ligase effectively (Von Hippel–Lindau protein functioning in human cells as E3 ubiquitin ligase), causing poly-ubiquitination and tau degradation in neuroblastoma cells and lowering tau levels in a mouse AD model. Lu et al. [193] reported PROTAC, which binds to the Keap1–Cul3 ubiquitin E3 ligase complex and induces tau degradation in different cell lines overexpressing tau. Silva et al. [194] combined a tau PET-probe with thalidomide analog, which binds cereblon (CRBN), a substrate-receptor for the E3 ubiquitin ligase CRL4^CRBN^. This PROTAC was able to induce the targeted degradation of aberrant tau in neurons derived from FTD patients. A similar approach, i.e., the use of thalidomide analogs to bind to cereblon, was presented by Kargbo RB [195], who synthesized several PROTAC compounds and found that they are capable of degrading hyperphosorylated tau and total tau proteins in htau-A152T and tau-P301L neurons. One of these compounds has the potential to cross the blood–brain barrier, as shown in the pharmacokinetic studies in mice.

It is worth mentioning that an analogous approach has been initiated regarding autophagy. Takahashi et al. [196] report that AUTAC (autophagy-targeting chimera), which consists of a degradation tag (guanine derivatives) and of a ligand providing target specificity, was able to degrade fragmented mitochondria.

## 7. Rescue from Tau Oligomers: Degradation or Polymerization?

Maintaining the physiological level and state of intracellular proteins—proteostasis—is essential for proper functioning of the cell. This applies particularly to neurons, whose function requires efficient trafficking of different cargos to and from soma to synapses and a continuous supply of energy required to uphold interneuronal signaling. Proteostasis is achieved by continuous protein synthesis, their restoration to a physiological state if possible, and if not possible—their degradation. The latter two processes are served by complex cellular machinery, which is collectively termed protein quality control (PQC) [144]. If these processes fail, the accumulation of toxic or inert proteins proceeds, resulting in malfunctioning, degeneration, and eventually neuron’s death. Many diseases are believed to result from imperfect proteostasis; among them are those with compromised tau turnover, termed tauopathies, AD being the prevailing one.

Intracellular aggregates of tau in the form of PHFs and NFTs are a hallmark of AD. Their presence and localization correlated better with the symptoms of AD and with the pathomorphological findings as the presence and localization of senile plagues being extracellular protein aggregates, mostly of Aβ [197]. For this reason, these tau aggregates were primarily believed to be neurotoxic and causal for AD or other neurodegenerative diseases.

However, some findings stripped NFTs away of their ominous reputation. It was found that NFT-bearing neurons in the visual cortex of human tau transgenic mice were completely functionally intact [198]. Based on quantitative data predicted from mathematical programs, it has been estimated that CA1 hippocampal neurons may stay alive about 20 years despite the presence of NFT [199]. Gómez-Isla et al. [200] revealed that the number of neurons lost in superior sulcus was manifold greater than the number of NFTs. This may mean that the presence of NFTs is not a necessary prerequisite for neuronal death [84] nor does its presence convey protection to neurons. However, the possibility that NFTs may impair cellular transport and lead slowly to neuronal death cannot be excluded.

The increasing number of new reports gradually developed a completely different view. It has be assumed that there is an inherent property of tau molecules toward acquiring multiple post-translational modifications, lending them the propensity to self-aggregation with age or due to different stress conditions [201]. The aggregation of tau seems to confer specific neurotoxicity to this protein [202]. Accumulating, though primarily indirect, evidence ascribed tau toxicity to short, soluble TauOs. In addition to indirect evidences implicating the toxicity of TauOs, there are also direct proofs of their selective toxicity in contrast to tau monomers and fibrillary moieties. Lasagna-Reeves et al. [92] found that oligomers of recombinant full-length human tau protein, but not monomers and fibrillary tau, were acutely neurotoxic in vivo after subcortical stereotaxic injection into mice brain. Of note, recombinant tau monomers obtained from *Escherichia coli* were unphosphorylated, not modified forms [203].

Therefore, the question arises as to whether there would be any adverse effects if post-translationally modified tau monomers were used. Liu et al. [204] showed that recombinant hyperphosphorylated tau (p-tau) tends to aggregate without an inducer and, unlike unmodified tau, it was cytotoxic to SH-SY5Y neuroblastoma and HEK 293T cells. These researchers used tau with a different degree of aggregation due to the different duration of aggregation. So, it is not clear whether they used p-tau monomers without some oligomers admixed. According to the results of Lasagna-Reeves et al. [92], as aggregation progressed, p-tau toxicity first increased and then decreased. Thus, p-tau toxicity appears to be related to the size of the tau aggregates, and this relationship is u-shaped. Another explanation for the lack of toxicity of monomers and filamentous tau may be that neurons can internalize low molecular weight tau aggregates and short filaments, while monomers, long fibrils, and long filaments do not internalize [205]. Nevertheless, the experiment by Lasagna-Reeves et al. [92] proved that TauOs (dimers/trimers) had a toxic effect when applied extracellularly. The importance of aggregate size was confirmed by Tian et al. [16], who found that tau trimers, but not monomers and dimers, were cytotoxic and led to the neuronal death.

Indirectly, the detrimental role of TauOs may be inferred from study, where a single injection of anti-tau oligomer-specific mouse monoclonal antibody (TOMA) reversed both locomotor and memory deficits in a mouse model of tauopathy. It also caused a reduction of TauOs but neither of phosphorylated NFTs nor monomeric tau [206]. Long-term treatment with TOMA effectively prevented the effects of intracerebral injection of pure AD brain-derived TauOs in wild-type and Htau mice [207].

In light of these findings, it seems likely that the transition from toxic soluble tau forms into less toxic or inert insoluble aggregates may rescue the cell. Cowan et al. [208] found that inhibiting tau GSK-3β not only rescued neurons from tau-induced dysfunction but also produced insoluble tau oligomers similar to granular tau oligomers (GTOs). Importantly, these GTOs-like structures were present in neurons in which cytoskeletal integrity was preserved, proving that this form is non-toxic. GTO, consisting of about 40 tau molecules, were first described by Maeda et al. [17] and interestingly, GTO presence in the frontal cortex was significantly increased in brains of persons without clinical symptoms of AD. Another salvage mechanism from the toxicity of soluble tau is described in an experiment in which caspases initiated the formation of tau tangles, and after their formation, the caspase activity declined, and the neurons remained alive [209].

Given the possibility that short TauOs have serious toxic effects and their rapid transition to neutral forms may be a salvation, it would be argued that AD is not the result of PHF and NFT accumulation, but rather the cause of the accumulation of these highly aggregated forms of tau, which may be a manifestation of neuroprotection. If it is indeed necessary to remove the soluble tau oligomers by converting them into insoluble granular oligomers and their further conversion to fibrillar structures, then PHF and NFT will only be the final step in the defense strategy. Once formed, NFTs can survive the breakdown of NFT-containing neurons and are detected in tissue as ghost tangles [200]. Thus, the true cause of appearance of NFT and PHF may be firstly, the specific for AD enhanced appearance of TauOs followed by their polymerization, and secondly, the inefficiency of protein degradation systems to remove aggregated forms of tau. When such a picture holds true, the strategy to combat tauopathies may or should rely firstly on enhancing the degradation of aberrantly modified tau monomers, which lost their physiological functions and turned into aggregation-prone toxic entities. Secondly, this strategy should encompass preventing tau from forming short oligomers, and thirdly, it should promote the enhancement of degradation of toxic TauOs.

Since it was realized that oligomeric tau can be a toxic species of tau, preventing tau oligomerization has become one of the goals of anti-AD therapy. As at present, we tend to believe that the degree of neurotoxicity is inversely proportional to the degree of oligomerization; blocking the first steps of formation of tau oligomers seems to be a desirable goal. It is less obvious whether preventing late stages of tau aggregation or the disassembly of tau polymers, such as fibrous structures, can also be considered beneficial. The advantages or disadvantages of breaking tau polymers should be weighed in connection with proteostasis—that is, whether the therapeutic effect increases the likelihood of purification of the products in the intracellular degradation process.

With the advent of belief in the deleterious role of oligomers, three strategies to prevent tau aggregation have been proposed by Guzman-Martinez et al. [210]. The first was to control tau phosphorylation by modulating the activity of protein kinases, in particular by inhibiting GSK-3β kinase [211]. The second mentioned strategy was treatment with methylthioninium chloride, also known as methylene blue (MB), and the third strategy recommends consuming natural phytocomplexes and polyphenolic compounds that are believed to inhibit tau fiber formation or even to disentangle highly aggregated filaments. The first method, although preclinically effective, has failed in many clinical trials for a variety of reasons. However, the search for new compounds continues [212]. In the case of MB, Soeda et al. [213] found that in vitro MB reduced the number of tau fibrils produced during its incubation with tau monomers. On the other hand, these authors found that MB increases the number of globular tau oligomers and intensifies the formation of low-mass tau oligomers: dimers, trimers—up to hexamers. These results, according to Soeda et al. [213], may explain the failure of the Phase III clinical trial (NCT01689246) published by Gauthier et al. [214]. This study did not show any effect in AD patients treated with the MB derivative leuco-methylthioninium bis (LMTM). However, further studies showed that the lowest dose of LMTM (4 mg/kg twice daily), originally intended as a placebo, was as effective as the much higher dose (100 mg/kg twice daily) in slowing cognitive decline and brain atrophy in patients with AD [215,216]. Similar results were obtained in patients with frontotemporal dementia with behavioral variants (bvFTD) [217]. This improvement may be due to the complex effect of MB on tau aggregation in vivo and/or to other properties of the compound. One of them is the autophagy-enhancing effect of MB [181], but MB also has other beneficial effects: it acts as an antioxidant, improves energy metabolism, reduces behavioral disturbances, and attenuates inflammation. Some of these beneficial effects may be due to the upregulation of the Nrf2/ARE genes (NF-E2 related factor 2/antioxidant response element), which play a role in antioxidant defense, prevent protein aggregation, and reduce inflammation [218]. Still, other beneficial effects of LMTM have been reported by Riedel et al. [219], such as an increase in acetylcholine levels in the hippocampus, glutamate release in synaptosomal preparations, and mitochondrial complex IV activity. It shows that MB/LMTM are in fact, though unintentionally, multi-target molecules. In the context of this review, an issue arises as to whether inhibition by MB of the tau transition from oligomers to polymers is actually beneficial or conversely harmful.

A similar property of increasing the level of tau oligomers at the expense of fibrous forms has been reported for cyanine, rhodamine, triarylmethine, and fulvic acid, which are a mixture of various polyphenolic acids [220]. It is now becoming increasingly important to believe that multi-target therapy, unlike the current dominant monotherapy, provides more promising results in the treatment of AD [221]. Thus, the concept of multi-targeting ligands (MTDLs) emerged [222]. The basic idea is to create compounds that are combinations of various pharmacophoric elements. Regarding the inhibition of tau aggregation, several types of molecules that act as dual inhibitors of both acetylcholine esterase and GSK-3β have been synthesized and tested in mice.

## 8. Conclusions

The central question emerging is whether soluble oligomers are really the most toxic form of tau. Answering this question will be helped by finding the answer to the question about the mechanisms of TauOs toxicity. It has to be realized that the term *soluble tau oligomers* is ill-defined, as there is a stunning variety of them. They may be aggregates of different tau monomers, which are possibly mutated as in FTDP-17, fragments of cleaved tau monomers, and in addition modified post-translationally in multiple ways. The relation between particular forms of TauOs and their toxicity has been not determined due to the lack of quantitative, high-resolution tools. It is hoped that the use of tau antibodies such as TOC1, T22, TOMA, and pS422, which selectively recognize dimers and higher order oligomers, will allow convincingly demonstrating the real toxic effect of tau oligomers on a number of cellular processes in tauopathies.

In the face of this seemingly unlimited variety of toxic entities, the interesting unified approach to the question of oligomers toxicity has been proposed by Kayed et al. [223]. They reason that there is a common conformation-dependent structure that is unique to soluble oligomers regardless of amino acid sequence, and that these oligomers share a common mechanism of toxicity. This common mechanism may rely on membrane permeabilization, which initiates a common group of detrimental processes, such as disturbed calcium homeostasis, ROS production, alteration of signaling pathways, and mitochondrial dysfunction [224]. On the other hand, given the specificity of tau functions, the oligomerization of tau may bring in addition tau-specific pathologic processes.

**Figure 2:**
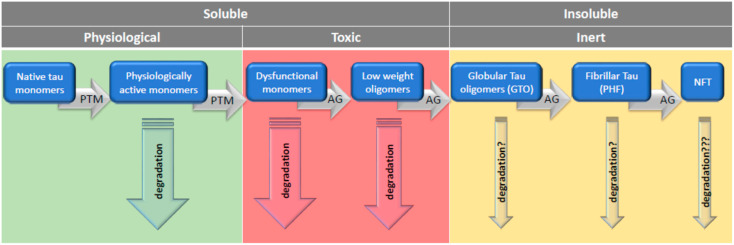
Illustration of the concept that tau protein undergoes the transition from native to physiologically active monomers thanks to the various post-translational modifications (PTM). At this point, it should be degraded by Protein Quality Control, paving the way to a newly synthetized tau monomer. Further PTMs may render the tau monomer toxic and/or aggregation prone. At this stage, it should be ultimately and promptly degraded. If this does not happen, tau monomers aggregate (AG), forming oligomers of low molecular weight (dimers, trimers, etc.), which are cytotoxic, soluble, and should be degraded as quickly as possible. Another way of cell rescue is furthering aggregation, which will decrease the solubility of tau oligomers and finally make them insoluble. It is believed that parallel with losing solubility, tau aggregates lose toxicity. It has been claimed that up from the point tau forms globular tau oligomers (GTO), consisting of about 40 tau molecules, tau aggregates are insoluble and inert. However, they may stand in the way of the transportation process within the cell. As the aggregation continues, tau assumes a fibrillar structure, forming first paired helical filaments (PHFs) and finally neurofibrillary tangles (NFTs). At every stage of transition from low weight oligomer to NFT, these tau forms may be degraded. However, in AD and in other tauopathies, the degradation of fibrillary tau forms is incomplete at best, leaving the cell with permanent deposits of highly aggregated NFTs. The optimal way of dealing with tau is to limit its presence within the physiological (green) zone, at best by combining increased degradation with aggregation inhibition. If, for any reason, tau encroaches the toxic (red) zone, it should either be degraded or transferred into the inert (yellow) zone. However, tau removal from the latter zone may be difficult and therefore incomplete due to the structure of aggregates and because of impaired and/or overburden autophagic pathways, mainly macroautophagic. Thus, at this stage, therapeutic measures should be focused on autophagy enhancement and optimization but not on inhibiting tau polymerization into inert forms.

Assuming that tau-soluble oligomers are the most toxic tau forms, then the optimal way is to avoid the transition of monomers into oligomers (Figure 2). This might be achieved by stimulating the degradation of no-aggregated tau forms with UPS, CMA, and eMI. Once toxic oligomers are produced, they might be promptly turned into insoluble tau aggregates and then degraded by macroautophagy. Such consideration points toward therapeutic strategies, as mentioned in this review i.e., modifying the chaperone–client protein interaction and intensifying the macrophagy pathway in various ways, which is the direction that seems really promising at present.

## Data Availability

Not applicable.
